# In Silico Analysis of Homologous Heterodimers of Cruzipain-Chagasin from Structural Models Built by Homology

**DOI:** 10.3390/ijms20061320

**Published:** 2019-03-15

**Authors:** Francisco Reyes-Espinosa, Alfredo Juárez-Saldivar, Isidro Palos, Verónica Herrera-Mayorga, Carlos García-Pérez, Gildardo Rivera

**Affiliations:** 1 Laboratorio de Biotecnología Farmacéutica, Centro de Biotecnología Genómica, Instituto Politécnico Nacional, Boulevard del Maestro s/n esq. Elías Piña, Col. Narciso Mendoza, Reynosa 88710, Mexico; ajuarezs1500@gmail.com (A.J.-S.); veronica_qfb@hotmail.com (V.H.-M.); 2 Unidad Académica Multidisciplinaria Reynosa-Rodhe, Universidad Autónoma Tamaulipas, Carr. Reynosa-San Fernando, Reynosa 88779, Mexico; isi_palos@hotmail.com; 3 Departamento de Ingeniería Bioquímica, Unidad Académica Multidisciplinaria Mante, Universidad Autónoma Tamaulipas, Blvd. Enrique Cárdenas González 1201, Mante 89840, Mexico; 4 Scientific Computing Research Unit, Helmholtz Zentrum München, 85764 Munich, Germany; carlos.garcia@helmholtz-muenchen.de

**Keywords:** cruzipain, chagasin-like, cystatin-like, molecular modelling, *Trypanosoma cruzi*

## Abstract

The present study gives an overview of the binding energetics of the homologous heterodimers of cruzipain−chagasin based on the binding energy (Δ*G*_b_) prediction obtained with FoldX. This analysis involves a total of 70 homologous models of the cruzipain−chagasin complex which were constructed by homology from the combinatory variation of nine papain-like cysteine peptidase structures and seven cysteine protease inhibitor structures (as chagasin-like and cystatin-like inhibitors). Only 32 systems have been evaluated experimentally, Δ*G*_b_^experimental^ values previously reported. Therefore, the result of the multiple analysis in terms of the thermodynamic parameters, are shown as relative energy |ΔΔ*G*| = |Δ*G*_b_^from^ ^FoldX^ − Δ*G*_b_^experimental^|. Nine models were identified that recorded |ΔΔG| < 1.3, five models to 2.8 > |ΔΔG| > 1.3 and the other 18 models, values of |ΔΔ*G*| > 2.8. The energetic analysis of the contribution of Δ*H* and Δ*S* to Δ*G*_b_ to the 14-molecular model presents a Δ*G*_b_ mostly Δ*H*-driven at neutral pH and at an ionic strength (*I*) of 0.15 M. The dependence of Δ*G*_b_(*I*,pH) at 298 K to the cruzipain−chagasin complex predicts a linear dependence of Δ*G*_b_(*I*). The computational protocol allowed the identification and prediction of thermodynamics binding energy parameters for cruzipain−chagasin-like heterodimers.

## 1. Introduction

The protozoan *Trypanosoma cruzi* (*T. cruzi*) is known as the etiologic agent of Chagas disease (CD) which is also termed American trypanosomiasis [1,2]. In endemic areas, the main vector of transmission of CD that has its natural ecosystem on the American continent is triatomine bugs (of the genus *Triatoma*). Other forms of CD transmission are congenital, by blood transfusion, by organ transplantation, orally (by food contaminated with parasites) or by laboratory accident [3,4]. With the purpose of treating CD, the scientific community has directed its efforts mainly in three lines of research: the search and design of new drugs, the design of biomarkers to test the effectiveness of drugs and techniques to diagnose the disease. To fulfil this purpose, a series of pharmacological targets have been identified (e.g., cruzipain, *trans*-sialidase, trypanothione reductase, triosephosphate isomerase, sterol 14-demethylase and tubulin, among others) as potential trypanocidal drugs.

Cruzipain (papain-like enzyme) is the major cysteine proteinase (CP) of *T. cruzi*. It belongs to the group of papain-like enzymes known as clan CA which is the most studied group of CP of parasitic protozoa [5,6]. The subcellular localization of cruzipain varies in different stages of the parasite’s biological cycle and is involved in penetration of the parasite and evasion of the host´s immune response. The parasitic protozoa contain cysteine peptidases (CP) that are crucial for a range of important biological processes [5]. These are involved in several stages of the parasite life cycle and thus exert substantial influence over host-parasite relationships, including metamorphosis, immune evasion and adaptation to specific hosts [6].

Natural protein structure inhibitors, known as cysteine protease inhibitors (CPI), regulate the catalytic activity of CP. These inhibitory proteins can be cystatin-like or chagasin-like. A complete description and classification of CP and CPI can be found in the MEROPS database (https://www.ebi.ac.uk/merops/ (accessed on February 2019)). A growing effort is currently being to design CPI from their endogenous and exogenous natural protein inhibitor (e.g., peptide derivatives as prodrugs and peptide mimetics) because of their high affinity [7,8] and specific CP inhibition of parasitic protozoa [9,10,11].

It is been nearly two decades since the first rationally designed small molecule drug (dorzolamide, an anti-glaucoma agent) was marketed [12]. Since then, the scientific community has made great efforts in the development of computational methods that allow the design of small molecules to produce biotherapeutics. This class of therapeutics includes proteins, peptides and nucleic acids that could more effectively combat drug resistance and even act when the disease is caused by a molecular deficiency [12].

On the other hand, in the field of bioinformatics, significant achievements have been made in the development of computational methods for modelling the protein−protein interaction [13,14], mainly in cases where a crystallographic complex is available with a potential to biologically bind to its target. Some methods such as molecular dynamics (MD) and other related methods can be used as routine computational tools for drug discovery. Limitations in the experimental techniques used to solve 3D structures make recurrence to theoretical molecular models necessary. When the appropriate templates are available, useful models can be built using some of the several software available today for this purpose. Homology modelling is a method of protein structure modelling. It is also called comparative modelling or sometimes template-based modelling (THM) [15,16]. Another technique used is the ligand-based methodology [17]. In recent decades, several 3D models of proteins have been constructed with these techniques. Some of these models have allowed a better understanding of function and structure and some are used as a guide for experimental works. They are also used in a wide variety of structural biology studies and in some cases provide successful molecular models for the design and discovery of new drugs [15,16,18].

The first complete in silico study of these systems, in the particular cysteine protease complex by homology modelling, was reported in 2011 by Tastan Bishop and Kroon [19]. Recent computational approaches are directed at understanding enzyme evolution from the perspective of protein structure, dynamics and promiscuity [20] as well as enzyme stability [21]. Currently, the FoldX program [22], which uses the empirical force field FoldX, is very useful for exploring point mutations in protein residues, in the design of high affinity binding proteins or in the study of recombinant proteins with potential therapeutic, diagnostic, industrial and basic science applications [23]. Therefore, we are interested in the study of these systems, mainly in the development of protein structure inhibitors of CP in pathogenic protozoa. Our objective is to elucidate the mechanism of PPIs using as a study method, the interaction of CP and CPI, proteins present in both protozoan pathogens and humans. In this context, we will refer to the CP−CPI complex as heterodimers formed by CP bound to the natural inhibitor with a protein structure. We carried out a literature search for homologous heterodimers of cruzipain−chagasin with inhibition constants (*K*_i_) reported in in vitro studies. These are showed in Table 1. These systems have a high sequence identity as well as a high structural similarity and share a common inhibition mechanism [19] and a *K*_i_ with a magnitude in the order of nM or less (Table 1). Therefore, their high specificity and the structural study of their binding make them attractive potentials for the natural design of inhibitors of the peptide structure of proteases, as a protein or as peptide inhibitors for use as drugs in the treatment of diseases caused by pathogenic protozoa.

In order to design new CPI against Chagas disease, we studied homologous heterodimers of cruzipain−chagasin; both proteins occur in *T. cruzi* protozoa. This analysis involved a total of 70 homologous heterodimers that were constructed from the combination of nine CP structures and seven natural CPI structures. The CP structures occur in protozoan pathogens (*T. cruzi*: cruzipain; *T. congolense*: congopain; *P. falciparum*: falcipain 2; and *L. mexicana*: LMCP and LMCP B) as well as in *human* (cath_B, cath_H, cath_L and cath_V) and plant (papain) organisms. The inhibitor structures involve natural CPI of protozoan pathogens (*T. cruzi*: chagasin; and *L. mexicana*: LEIME) as well as cystatin of *human* (cyst_A, cyst_B and cyst_C), *coturnix* (Cj_cyst) and *gallus* (Gg_cyst) organisms. We analysed papain-like structures such as cystein-protease (CP) biding to the natural inhibitor cystein-protease (CPI) and performed an in silico analysis of their dependence of Δ*G*_b_ with the variation of pH, temperature and ionic strength.

## 2. Results

**Figure 1:**
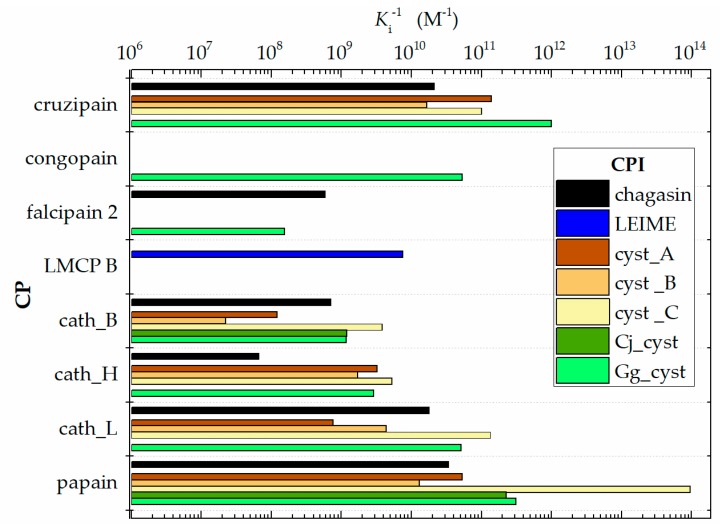
Graphical comparison of (*K*_i_)^−1^ of homologous heterodimers of cruzipain−chagasin. (Data obtained from Table 1).

There is little published information on *K*_i_ values determined by spectroscopic methods or other instrumental analysis methods of homologs of the cuzipain−chagasin complex (Table 1). These originate from different sources and time periods, have been normalized and can be truly compared. Only 32 systems have been evaluated experimentally, Δ*G*_b_^experimental^ values previously reported. However, using the information obtained from Δ*G*_b_ prediction of the systems under study and the knowledge of the structural levels (mainly residue sequence information and crystallographic structures of free and complexed protein) of the homologous heterodimers of cuzipain−chagasin, it was feasible to carry out the present study. In order to obtain the optimal visual appreciation of *K*_i_ values obtained in in vitro experiments previously reported, we present in Figure 1 the (*K*_i_)^−1^ profile of homologs of the cuzipain−chagasin complex. 

### 2.1. Heterodimer Models Constructed by Homology Modeling

**Figure 2:**
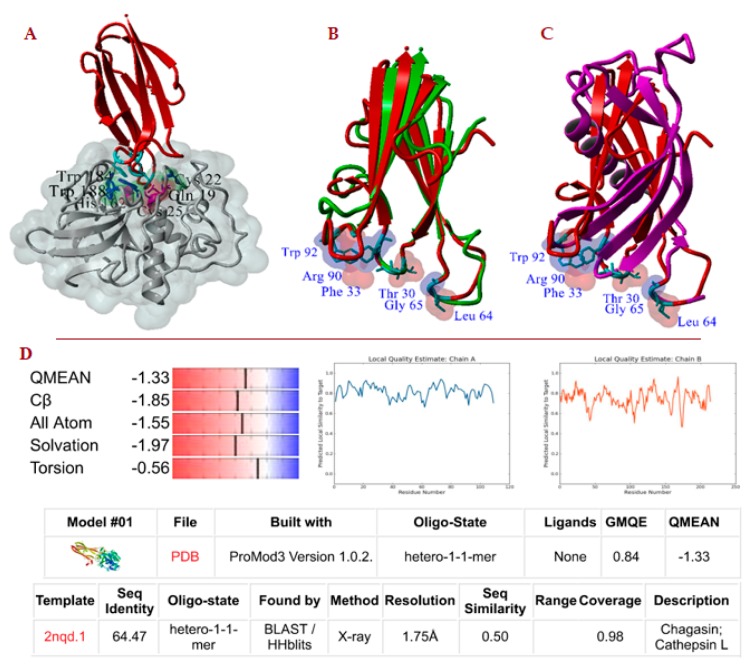
Molecular models constructed by homology. (**A**) cruzipain−chagasin complex: Chain in red, chagasin structure; chain in grey, cruzipain structure. (**B**,**C**) 3D alignment of molecular models of LEIME (chain in green) and Gg_cystain (chain in magenta) on chagasin (chain in red) -numbering of chagasin structure-. (**D**) Typical SWISS-MODEL homology modelling report (e.g., cruzipain-chagasin complex).

Seventy molecular models, homologous cruzipain-chagasin heterodimers (e.g., Figure 2A), were constructed by homology modelling using the SWISS-MODEL server [34]. The details of the templates used are presented in Table 2. Two model quality evaluation parameters are showed. The first score refers to the global model quality estimation (QMQE) from the model building which is presented in a SWISS-MODEL homology modelling report [34] (e.g., Figure 2D) and the second, the protein-protein complex evaluation that is calculated by the PROCOS program [35]. All constructed models that registered a QMQE score > 0.72 or a PROCOS score > 0.75 were optimized. These models were refined with the GalaxyRefineComplex program by interface repacking, which successfully improved homology model structures. This refinement method allows flexibility at the protein interface and in the overall structure to capture conformational changes that occur upon binding [36]. Δ*G*_b_ was calculated by FoldX, which uses the empirical force field FoldX [22]. Previously, to evaluate energy, the Repair-FoldX tool was used to avoid the presence of incompatibilities between the CP and CPI structure.

Validation of the computational methodology was carried out by evaluating the RMSD parameter of the nine molecular models obtained by homology, using as a reference their respective crystal complex heterodimer structures obtained from the Protein Data Bank as well as the PROCOS score [35]. This comparison of results is showed in Table 3. A more detailed analysis of the QMQE score and the PROCOS score of the 70 molecular models constructed by homology is presented in Table 2. We also performed an analysis of the composition of the residues in the interface of the PDB structures. The corresponding sequence alignment and the conserved residues identified in the interface of homologous heterodimers of cuzipain−chagasin are presented in Appendix A.

### 2.2. ΔG_b_ Prediction of Homologs of the Cruzipain−Chagasin Complex

**Figure 3:**
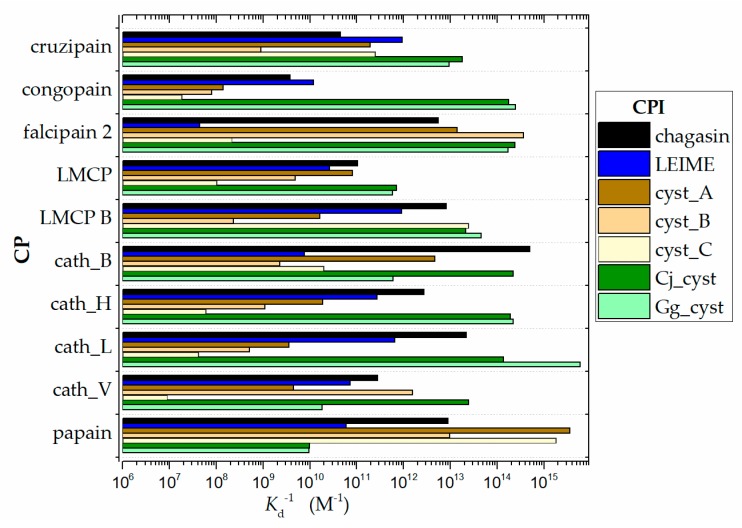
Data profile of affinity (*K*_a_ = *K*_d_^−1^) from analysis of the 70 homologous heterodimers of cruzipain−chagasin, which were constructed by homology; (*K*_d_)^−1^ data grouped by CP. *K*_a_ was calculated from Δ*G.* Δ*G* was evaluated with the FoldX program: pH 7.0 at T 298 K and *I* = 0.15 M.

The predicted Δ*G*_b_ values obtained from the 70 models built by homology for the different systems studied are showed in a range of −21.5 to −9.5 Kcal/mol. In terms of affinity as *K*_a_ = (*K*_d_)^−1^ (see Materials and Methods), these results recorded a moderate affinity (10^7^ to 10^12^ M^−1^) and a high affinity (10^12^ to 10^15^ M^−1^). The (*K*_d_)^−1^ data profile is shown in Figure 3.

### 2.3. Energetic Analysis

**Figure 4:**
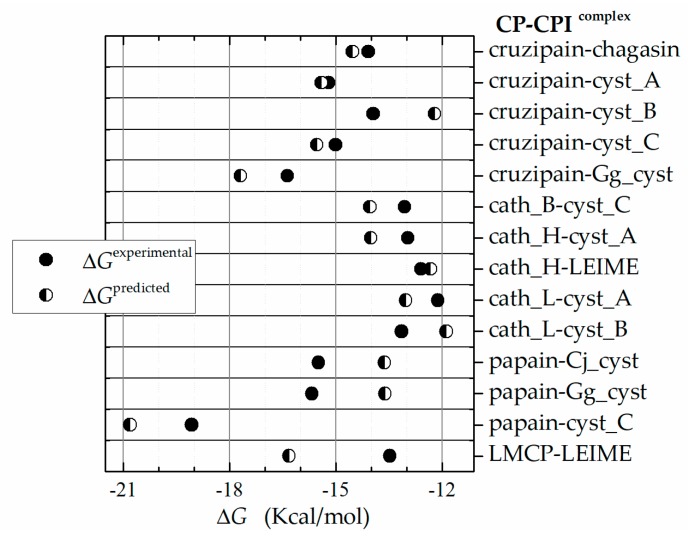
Analysis in terms of relative binding energy. Δ*G*_b_^predicted^, obtained from FoldX, Δ*G*_b_^experimental^, obtained from *K*_i_ (Table 1). A Graph representation of competitive inhibition of cruzipain with chagasin inhibitor homologs (|ΔΔ*G^i^*| = |Δ*G*_b_^from FoldX^ − Δ*G*_b_^experimental^|). B Graphical record of energy Δ*G.*

In order to carry out a multiple analysis of the 70 models of homologous heterodimers of cuzipain−chagasin, we calculated ΔΔ*G* and considered the presence of two ligands that bind to the same receptor. Therefore, the multiple analysis in terms of thermodynamic parameters was performed as |ΔΔ*G*| = |Δ*G*_b_^from FoldX^ − Δ*G*_b_^from Ki^| in absolute value (see Section 4). In this analysis, nine models were identified that recorded |ΔΔG| < 1.3 (dimer systems as cruzipain in complex with chagasin, cyst_A, cyst_C and Gg_cyst and also cath_H−LEIME, cath_H−cyst_A, cath_L−cyst_A, cath_L−cyst_B and cath_B−cyst_C) and five models to 2.8 > |ΔΔG| > 1.3 (systems as cruzipain−cyst_B, papain−cyst_C, LMCP−LEIME, papain−Cj_cyst and papain−Gg_cyst) (see Figure 4). The other twenty models for comparison showed a tendency or profile according to the behaviour between the systems analyzed but not in magnitude (|ΔΔG| > 2.8).

**Figure 5:**
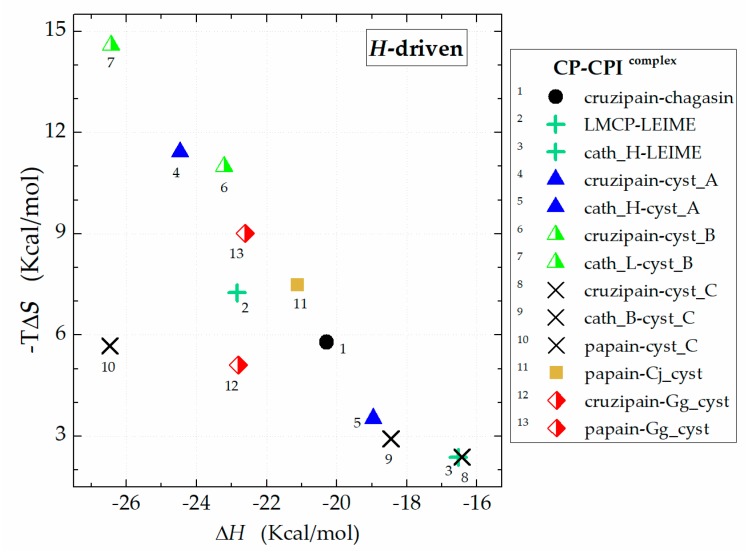
Data profile of the contribution of enthalpy (Δ*H*) and entropy (−TΔ*S*) to Δ*G*_b_.

The Gibbs equation is defined as Δ*G*_b_ = Δ*H* − *T*Δ*S* and involves two components that contribute energy to the equilibrium, enthalpy (Δ*H*) and entropy (Δ*S*). The predicted Δ*G*_b_ was obtained at the temperatures of 298, 313 and 323 K. Then, a van‘t Hoff plots analysis was performed to obtain the thermodynamic terms (Δ*H* and Δ*S*). Thus, the Δ*G_b_* expressed in terms of the contribution of Δ*H* and Δ*S* is showed in Figure 5. The magnitude of the contribution of Δ*H* and ΔS to Δ*G* are presented in Appendix B.

**Figure 6:**
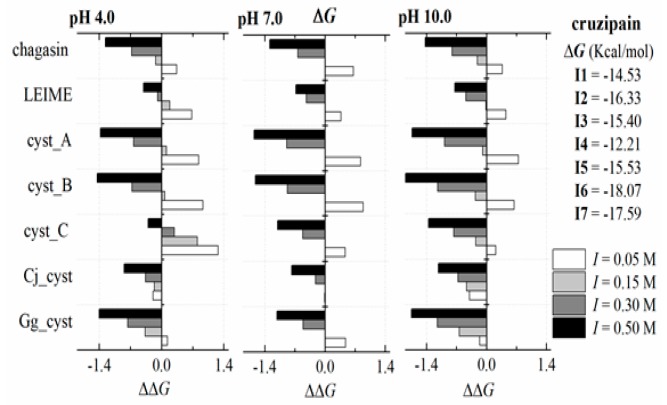
Cruzipain−CPIs complex. Δ*G*, free energy; ΔΔ*G* = Δ*G*_b_ − Δ*G* relative binding energy, where Δ*G*_b_ (*I*, pH) at 273 K and Δ*G* reference was predicted at 278 K, pH 7.0 and *I* 0.15 M. Inhibitors: I1, chagasin; I2, LEIME; I3, cyst_A; I4, cyst_B; I5, cyst_C; I6, Cj_cyst; I7, Gg_cyst.

Finally, we present the results of a multiple analysis of the 14 models of homologous heterodimers of cuzipain−chagasin and their Δ*G*_b_(*I*, pH) profiles based on ΔΔ*G*_b_ (relative binding energy). These studies are present in Appendix B. The typical result of this analysis for the particular case of cruzipain in interaction with the seven inhibitors is shown in Figure 6.

## 3. Discussion

### 3.1. Analysis of K_i_ Values Obtained in In Vitro Experiments (Reported in the Literature)

Homologous cruzipain-chagasin complexes previously reported, this shows *K_i_* records in the range of nM to fM (Table 1). The cruzipain-chagasin and cruzipain-cyst_B complexes recorded a *K*_i_ of around 0.045 nM. In reference to the cruzipain-cyst_A, cruzipain-cyst_C cruzipain-Gg_cyst complexes have an order of magnitude in *K*_i_ less than that registered in the cruzipain-chagasin complex. It highlights a particular interest other systems such as cath_L-cyst_C, papain-cyst_C and papain-Gg_cyst complex due to its low registered *K*_i_.

### 3.2. Molecular Models Constructed by Homology Modeling and Methodology Validation

The methodology implemented in the development of this work was validated with satisfactory results. The PROCOS results showed a probability-like measure to be a native composition interface for the 70 molecular models analysed. The PROCOS scores collected from the nine PDB structure analysis were values of 0.47 to 1.0 (66.7% of values between 0.8 and 1.0) and 0.59 to 0.99 for the respective molecular models constructed by homology (Table 2). Values with the lowest probability may be an implication that shows very high van der Waals energies [36] such as those of the 3CBK, 3KSE and 3KFQ complex. The global analysis of the molecular model constructed by homology showed a PROCOS score of 75% for values of 0.75 to 1.0. We considered, according to the validation results, that in low scores there is a presence of strong nonspecific interactions due to the presence of aromatic residues at the interface; only 33.3% of models constructed were refined with the GalaxyRefineComplex program (see Table 2). An extensive analysis of the interface of cysteine protease−cystatin structures, such as crystal structures (PDB ID: 1STF, 3K9M, 1NB5, 3KSE and 3KFQ) and two molecular models (chymo−Gg_cyst and papain−Gg_cyst complex) has been presented previously [38].

### 3.3. Analysis of ΔG_b_ Prediction Values

**Figure 7:**
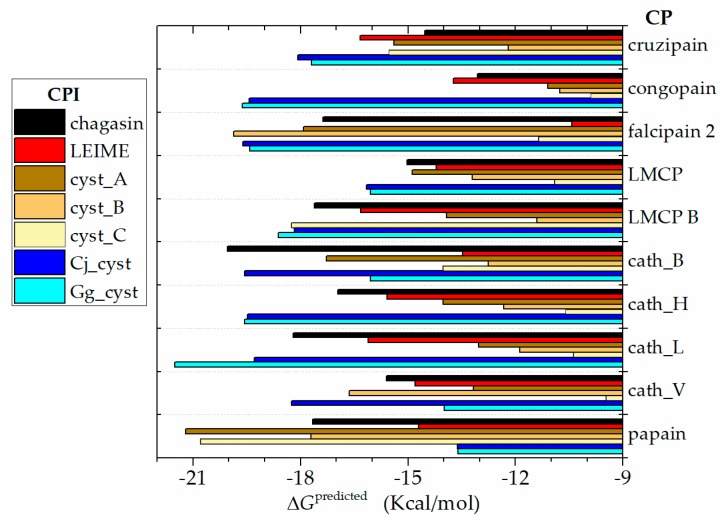
Data profile of Δ*G*_b_ prediction of the 70 homologous heterodimer of cruzipain−chagasin that were constructed by homology. Δ*G* was evaluated with the FoldX program: pH 7.0 at T 298 K and *I* = 0.15 M.

The analysis of Δ*G*_b_ from cruzipain-CPIs complex (Figure 7) reveals that CPI proteins from protozoan pathogens both cruzipain-chagasin and cruzipain-LEIME complex (−14.5 and −16.3 Kcal/mol, respectively) register a Δ*G*_b_ magnitude close to cruzipain-cyst_A and cruzipain-cyst_C (both CPIs of human) of −15.4 Kcal/mol. While the cruzipain-cyst_B complex recorded a higher Δ*G*_b_ value (−12.2 Kcal/mol) compared to that obtained for cruzipain-chagasin complex. Whereas the inhibitors Cj_cyst and Gg_cyst register a considerably lower Δ*G*_b_ (−18.1 and −17.7 Kcal/mol, respectively) than cruzipain- chagasin complex. Other important correlations in the CP-CPI complex study may emerge from a more detailed analysis of the predictions presented in Figure 7. Of our particular interest is the prediction of a high affinity of chagasin for cathepsins human -cath_B, cath_H, cath_L and cath_V- (Δ*G*_b_ of −15.6 to −20.0 Kcal/mol) as well as bird cystatins (Cj_cyst and Gg_cyst) for cathepsins human and CP from protozoan pathogens (e.g., cruzipain, congopain, falcipain 2, LMCP and LMCP B). Plant CPs such as papain-chagasin complex showed interesting lower Δ*G*_b_ magnitudes (−17.3 Kcal/mol) compared to cruzipain-chagasin complex.

The results presented in the FoldX predictions (Figure 3) with the experimental data (Figure 1) are not comparable; conceptually *K*_i_ is different from *K*_d_. Therefore, the presented magnitudes of *K*_i_ as experimental data do not need to be of the same order of magnitude as *K*_d_ obtained by the predictions of FoldX. In a reversible bimolecular mechanism of a simple one-step model, the *K*_d_ and the equilibrium association constant (*K*_a_) are reciprocally related (*K*_a_ = *K*_d_^−1^) [38]. On the other hand, the inhibition constant (*K*_i_) can be determined or approximated (*K*_i_^app^) with various enzymatic kinetics from data obtained in an enzyme inhibition experiment (for example, methods that use a nonlinear expression more accurately estimate *K*_i_ as well as values for *K*_M_ and *V*_MAX_ or the linearized approach of Dixon [39]). In ligand binding studies and under controlled conditions, *K*_i_ can be calculated with the Cheng and Proof equation, where it is assumed that *K*_i_ ≈ *K*_d_ = (*K*_a_^−1^) [40].

Performing a global analysis of results in terms of Δ*G*_b_, there is no clear trend in the behaviour of the energy magnitudes obtained. This variability of magnitudes recorded for Δ*G*_b_ in the structural homologs of the cruzipain−chagasin complex is inherent in the composition of the residues that constitute their respective interfaces. The energy analysis of the contribution of Δ*H* and Δ*S* to Δ*G*_b_ of the 70-molecular model of homologs of the cruzipain−chagasin complex presents a Δ*G*_b_ is mostly Δ*H*-driven (Figure 5) at neutral pH and at an ionic strength (*I*) of 0.15 M.

The dependence of Δ*G*_b_ (*I,* pH) at 298 K to the cruzipain−chagasin complex at three pH values (pH 4.0, 7.0 and 10.0) predicts a linear dependence of Δ*G*_b_ (*I*) with R^2^ values equal to 0.9977 (at acid pH), 0.9999 (at neutral pH) and 0.9997 (at alkali pH). Similar results (R^2^ values > 0.99) were recorded in systems such as cyst_A, cyst_B, cyst_C and Gg_cyst in complex with cruzipain. Cruzipain−LEIME and cruzipain−Cj_cyst systems recorded R^2^ values > 0.77 and >0.98, respectively (Figure 6). Other systems of homologous heterodimers of cruzipain−chagasin presented similar results (see Appendix B). 

Papain-like CP play an important role in the regulation of biological cycles of all organisms. Endogenous protein structure inhibitors such as chagasin-like and cystatin-like regulate its biological function. We can appreciate in Figure 1 that cysteine proteases of the protozoan pathogen have greater specificity and a high affinity for their human endogen inhibitors (cystatin-like) compared to their natural inhibitor (chagasin). Regarding the natural chagasin-like inhibitors such as chagasin and LEIME, the predicted Δ*G*_b_ reveals a high affinity for human cathepsins much better than human cystatins (Figure 7).

**Figure 8:**
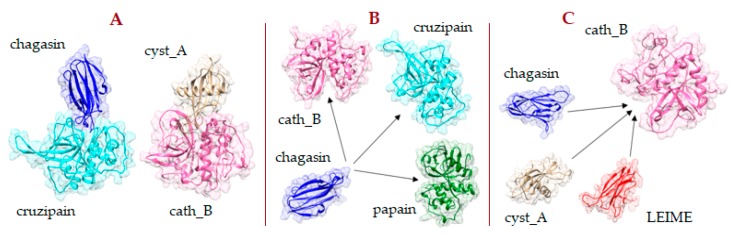
Putative enzyme−inhibitor interaction between agonists−antagonists in three possible scenarios that could occur in the treatment of disease from pathogenic protozoa. (**A**) Enzyme−natural inhibitor as a like-agonist interaction; (**B**,**C**) Enzyme−natural inhibitor as a like-antagonist interaction. Alternative interaction to regulate the biological metabolism in the human organism by an exogenous agent such as an enzyme or natural inhibitor.

Considering the results analyzed, it is possible to formulate two hypothetical events that could occur simultaneously in the invasive process of the pathogenic protozoan (Figure 8). The first favours the metabolism of the pathogenic protozoan where endogenous CPI of the human (^CPI_hum^) is not able to inhibit the CP of the protozoan pathogen (^CP_pp^); that is, Δ*G*_b_^CP_pp-CPI_pp^ << Δ*G*_b_^CP_pp-CPI_hum^. The second event occurs in favour of the parasite defense mechanism due to the presence of endogenous CPI inhibitors of pathogenic protozoan (^CPI_pp^) with a high affinity that can inactivate human CP (^CP_hum^); that is Δ*G*_b_^CP_hum-CPI_pp^ << Δ*G*_b_^CP_hum-CPI_hum^. At this moment, the putative situation that we have cited is of great interest to the scientific community and innovative research is being developed in this respect to continue discovering and characterizing new natural inhibitors of this protein structure [7].

The agonist interactions between CP and CPI allow correct metabolic function of the living organism. In the infective process of a human organism by a pathogenic protozoan, the presence of both CP and exogenous PCI (from the protozoan pathogen) is a determinant that causes the disease because the defense mechanism of the human organism is affected or interrupted. A potential solution to these antagonistic interactions that could be applied in the pharmacological treatment of the disease caused by pathogenic protozoa is the exogenous insertion of natural plant CPI or CP [7].

In agreement with the previously reported information about the experimental *K*_i_ heterodimer homologs of cruzipain−chagasin (Table 1) and the respective analysis presented in this work, we suggest that the presence of highly specific cross-linkers and chagasin for homologous agonists (cathepsins and cystatins) in humans is a key point of the evolutionary persistence of Chagas disease. The development of new CPIs with a protein structure and peptide inhibitors derived from these protein structures is based on the study of the interaction of natural PCI such as chagasin-like and cystatin-like inhibitors in order to develop new antiparasitic drugs that have non-toxic properties, high specificity, biological selectivity and potent and reversible inhibition.

## 4. Materials and Methods

**Figure 9:**
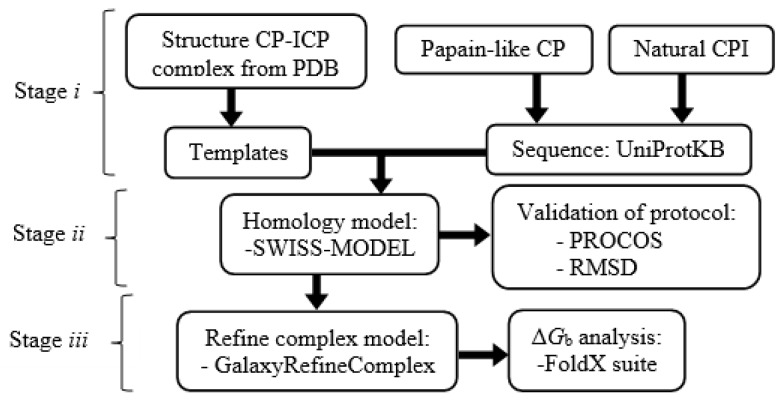
Methodology developed to predict the binding energy (Δ*G*_b_) of heterodimers modelled by structural homology.

The protocol used to study the 70 homologous heterodimers of cruzipain−chagasin was divided into three stages (the methodology is presented in Figure 9): (*i*) Identification templates in RCSB PDB and alignment of sequences obtained from UniProtKB; (*ii*) Model building with the SWISS MODEL [34] and refinement of model interfaces with GalaxyRefineComplex [36] and stage (*iii*) Analysis of binding energy (Δ*G*_b_) with the FoldX suite [22].

### 4.1. Preparation and Analysis of Sequences

Ten crystal complex structures (ID PDB: 1YVB, 2OUL, 3CBK, 3CBK, 3K9M, 2NQD, 3KSE, 3KFQ and 1STF) and five free protein structures (ID PDB: 1ME3, 1YAL, 1PPO, 3GAX and 2C34) were selected from the RCSB PDB (www.rcsb.org (accessed on February 2019)) [37] as templates (Table 4) for the study of homologous heterodimers of the cruzipain−chagasin complex. All structures were refined by subtracting water molecules and external chains. Energy minimization was performed using the YASARA force field [41]. These PDB structures include CP and CPI that occur in organisms such as protozoan pathogens, plants and humans. The respective sequences were recovered with their accession number from UniProt [42]. The sequences were aligned with those reported in the crystal structures and cut to the same length; these protein sequences were used to build the structure models.

### 4.2. Structural Modeling by Homology

Structural models of homologous heterodimers were generated with the SWISS-MODEL server [34], an automated protein homology-modelling server. Each pair of the protein sequences that form the heterodimer (previously prepared) was loaded into the SWISS-MODEL server using the template mode [34]. The interfaces of all the structures generated were refined with the GalaxyRefineComplex program [36] installed in a computer with a 3.4 GHz Intel Core i7 processor and 23.5 GB RAM and with Linux Mint 17.3 as the operating system. The generated structural models were validated by measuring two parameters. The first was the RMSD (root-mean-square deviation) of the models obtained with respect to the crystallographic structures reported. The second was the evaluation of the probability of amino acids in the interface of the models. This measurement was made with the PROCOS server [35].

### 4.3. Evaluation of Heterodimer Models and ΔG_b_ Analysis

Δ*G*_b_ was evaluated in all models built with the FoldX program [22]. FoldX uses the 3D structure to calculate the energy. The empirical force field algorithm is based on free energy (Δ*G*) terms aiming to calculate the Δ*G* in Kcal/mol. The FoldX energy function includes terms as van der Waals, solvation (polar and hydrophobic), electrostatic, hydrogen bonds and so forth [43,44]. Other relevant aspects about FoldX program are brief described in official website. Available online: http://foldxsuite.crg.eu/products (accessed on February 2019) [45]. We did not perform a test case of the modelling/energetics from FoldX program against MD simulation [43]. 

In order to identify a modulation of Δ*G*_b_(pH) due to changes in the electrostatic energy [38], the dependence of Δ*G*_b_ was studied with a pH variation of 3.0 (acid pH), 7.0 (neutral pH) and 10.0 (alkali pH), at temperature of 298 K and at an ionic strength (*I*) of 0.15 M. The dependence of Δ*G*_b_(*I*, pH) (at ionic strengths of 0.05, 0.15, 3.0 and 5.0 M) was studied to three pH values (pHs of 3.0, 7.0 and 10.0), at temperature of 298 K. Also, the dependence of Δ*G*_b_(T) was studied at temperatures of 298, 313 and 323 K, pH 7.0 (at neutral pH) and at an ionic strength of 0.15 M. Prior to the execution of the simulation, the variations of the parameters of pH, temperature and ionic strength were established in the commands used to operate FoldX, these are RepairPDB command and AnalyseComplex command [45]. 

In order to obtain the thermodynamic terms of Gibbs equation (Δ*G* = Δ*H* − TΔ*S*), enthalpy (Δ*H*) and entropy (ΔS), we used the method based on Δ*G*_b_ prediction at different temperatures combined with van‘t Hoff plot analyses (ln*K* = −Δ*H*/(RT) + Δ*S*/R; *K*, equilibrium constant and R, ideal gas constant) to obtain the thermodynamic terms of Gibbs equation (Δ*G* = Δ*H* − TΔ*S*), enthalpy (Δ*H*) and entropy (Δ*S*). Therefore, we proceeded to generate a typical graph 1/T versus ln*K*, the slope = −Δ*H*/R and intercept = Δ*S*/R. Solving the linear equation resulting from the linear fitting, we obtained Δ*H* = (slope) (−R) and Δ*S* = (intercept) (R).

According to the equation in the equilibrium system, we can define free energy, Δ*G*_b_ = −RTln*K*_a_ = RTln*K*_d_; where R is a gas constant, T is temperature and *K*_a_ is a binding constant (*K*_a_ = 1/*K*_d_, where *K*_d_ is dissociation constant) [12,38,40]. ΔΔ*G* is defined as ΔΔ*G* = Δ*G*_b_^II^ − Δ*G*_b_^I^ = RT ln(*K*_d_^II^/*K*_d_^I^). The term ΔΔ*G* is a thermodynamic parameter that allows us to identify the Δ*G*_b_ difference present in a system under study (Δ*G*_b_^I^) in relation to a reference system (Δ*G*_b_^II^). It is well known that if it is present, a ten-fold difference in *K*_d_s at 298 K corresponds to a ΔΔ*G* difference of 1.34 Kcal/mol. Therefore, a ΔΔ*G* difference of 1 Kcal/mol at 298 K corresponds to a 5.4-fold difference in *K*_d_s. In both hypothetical situations the binding of ligand 2 is weaker than that of ligand 1. In the present work, we use ΔΔ*G* to compare the Δ*G*_b_ recorded in in silico study with data reported previously.

## 5. Conclusions

In the present in silico study, we can suggest that from the scaffolding of the interaction of bird cystatins (Cj_cyst and Gg_cyst), it is possible to design or propose natural inhibitors with a high affinity for protozoan proteases. Therefore, it is possible to rationally design both highly selective antagonistic proteins to CP and CPI from protozoan pathogens. The interface composition is decisive to modulate Δ*G*_b_. Chagasin-like and cystatin-like structures in interaction with cruzipain homologs showed that Δ*G*_b_ is mostly Δ*H*-driven at neutral pH and at an ionic strength (*I*) of 0.15 M. Because of their wide structural stability to pH, ionic strength (*I*) and temperature changes (previously reported in in vitro studies by other researchers) and according to the results presented, we propose Cj_cyst and Gg_cyst as excellent candidates and desirable scaffolding for the design of new cruzipain inhibitors derived from peptide structure. The computational protocol based on Homology Modelling and FoldX predictions allowed the identification and prediction of thermodynamics parameters of binding energy for cruzipain−chagasin-like heterodimers.

## Figures and Tables

**Table 1:** Experimental inhibition constant experimental (*K*_i_, nM) of homologous heterodimers of cruzipain−chagasin.

Enzyme	Natural Inhibitor Cystein-Protease						
	Chagasin	LEIME	cyst_A	cyst _B	cyst _C	Cj_cyst	Gg_cyst
cruzipain	0.018 ^d,b^		0.007 ^a,k^	0.060 ^a^	0.01 ^a,h^		0.001 ^a,k^
	0.076 ^i^				0.005 ^d^		
congopain							0.019 ^f^
falcipain 2	1.70 ^b,c^						6.50 ^c^
LMCP B		0.133 ^j^					
cath_B	0.93 ^b^		8.20 ^a^	73.00 ^a^	0.27 ^a,d^	0.828 ^g^	0.81 ^e^
	1.90 ^d^			16.00 ^b^	0.26 ^e^		0.047 ^g^
cath_H	15.00 ^c^		0.31 ^a^	0.58 ^a^	0.28 ^a^		0.06 ^a^
					0.10 ^e^		0.63 ^c^
cath_L	0.039 ^d,b^		1.30 ^a^	0.23 ^a^	<0.005 ^a,d^		0.019 ^a,e^
papain	0.036 ^b^		0.019 ^a^	0.12 ^a^	0.00001 ^a,d^	0.004 ^g^	0.005 ^a,e^
	0.023 ^d^			0.03 ^b^			0.0014 ^g^

^a^ Turk et al. [24]; ^b^ Redzynia et al. [25]; ^c^ Wang et al. [26]; ^d^ Ljunggren et al. [27]; ^e^ Rowan et al. [28]; ^f^ Chagas et al. [29]; ^g^ Gerhartz et al. [30]; ^h^ Lima et al. [31]; ^i^ Dos Reis et al. [32]; ^j^ Smith et al. [11]; ^k^ Stoka et al. [33].

**Table 2:** Model quality estimation.

**Model**	**E1_I1**	**E2_I1**	**E3_I1**	**E4_I1**	**E5_I1**	**E6_I1**	**E7_I1**	**E8_I1**	**E9_I1**	**E10_I1**
Template ^a^	2NQD	2NQD	2NQD	2NQD	2NQD	3CBK	2NQD	2NQD	2NQD	3E1Z
GMQE ^b^	0.84	0.83	0.78	0.84	0.84	0.99	0.84	0.98	0.97	0.99
PROCOS ^c^	0.91	0.97	0.97	1.00	0.97	0.78	0.97	0.99	1.00	0.69
**Model**	**E1_I2**	**E2_I2**	**E3_I2**	**E4_I2**	**E5_I2**	**E6_I2**	**E7_I2**	**E8_I2**	**E9_I2**	**E10_I2**
Template ^a^	2NQD	2NQD	3PNR	2NQD	2NQD	3CBK	2NQD	2NQD	2NQD	3E1Z
GMQE ^b^	0.73	0.73	0.84	0.73	0.73	0.89	0.73	0.87	0.86	0.87
PROCOS ^c^	0.97	0.97	0.61	0.99	0.97	0.62	0.99	0.97	1.0	0.97
**Model**	**E1_I3**	**E2_I3**	**E3_I3**	**E4_I3**	**E5_I3**	**E6_I3**	**E7_I3**	**E8_I3**	**E9_I3**	**E10_I3**
Template ^a^	3KSE	3KSE	3K9M	3KSE	3KSE	3K9M	1NB5	3KSE	3KSE	3K9M
GMQE ^b^	0.83	0.83	0.71	0.83	0.83	0.99	0.98	0.98	0.97	0.75
PROCOS ^c^	0.59	0.86	0.97	0.70	0.82	0.97	0.97	0.63	0.59	0.67
**Model**	**E1_I4**	**E2_I4**	**E3_I4**	**E4_I4**	**E5_I4**	**E6_I4**	**E7_I4**	**E8_I4**	**E9_I4**	**E10_I4**
Template ^a^	3KSE	3KSE	3K9M	3KSE	3KSE	3K9M	1NB5	3KSE	3KSE	3K9M
GMQE ^b^	0.77	0.77	0.66	0.76	0.77	0.93	0.92	0.92	0.91	0.72
PROCOS ^c^	0.86	0.97	0.97	0.91	0.98	0.78	0.97	0.64	0.82	0.82
**Model**	**E1_I5**	**E2_I5**	**E3_I5**	**E4_I5**	**E5_I5**	**E6_I5**	**E7_I5**	**E8_I5**	**E9_I5**	**E10_I5**
Template ^a^	1YVB	1YVB	1YVB	1YVB	1YVB	1YVB	1YVB	1YVB	1YVB	1YVB
GMQE ^b^	0.74	0.75	0.92	0.73	0.74	0.64	0.74	0.74	0.74	0.74
PROCOS ^c^	0.94	0.97	0.86	0.67	0.69	0.91	0.97	0.91	0.69	0.67
**Model**	**E1_I6**	**E2_I6**	**E3_I6**	**E4_I6**	**E5_I6**	**E6_I6**	**E7_I6**	**E8_I6**	**E9_I6**	**E10_I6**
Template ^a^	1YVB	1YVB	1YVB	1YVB	1YVB	1YVB	1YVB	1YVB	1YVB	1YVB
GMQE ^b^	0.82	0.82	0.98	0.80	0.81	0.71	0.82	0.82	0.82	0.82
PROCOS ^c^	0.97	0.97	1.00	0.97	0.97	0.97	0.97	0.97	0.99	0.82
**Model**	**E1_I7**	**E2_I7**	**E3_I7**	**E4_I7**	**E5_I7**	**E6_I7**	**E7_I7**	**E8_I7**	**E9_I7**	**E10_I7**
Template ^a^	1YVB	1YVB	1YVB	1YVB	1YVB	1YVB	1YVB	1YVB	1YVB	1YVB
GMQE ^b^	0.81	0.81	0.99	0.81	0.81	0.82	0.82	0.71	0.82	0.82
PROCOS ^c^	0.97	0.97	0.98	0.97	0.97	0.91	0.99	0.97	0.99	0.94

^a^ ID complex from Protein Data Bank. ^b^ Global model quality estimation [1]. ^c^ Interface evaluation of protein–protein complex [2]. Protease structures: E1, cruzipain; E2, congopain; E3, falcipain 2; E4, LMCP; E5, LMCP B; E6, cath_B; E7, cath_H; E8, cath_L; E9, cath_V; E10, papain. Inhibitor structures: I1, chagasin; I2, LEIME; I3, cyst_A; I4, cyst_B; I5, cyst_C; I6, Cj_cyst; I7, Gg_cyst.

**Table 3:** Validation dataset protocol for building models.

PDB ID ^a^	SM_HM	RMSD (Å)	Sequence Identity (%)	Scores PROCOS	
				PDB	SM HM
2OUL	falcipain−chagasin	1.22	100.0	0.97	0.97
1YVB	falcipain−Gg_cyst	0.42	100.0	0.98	0.99
3CBK	cath_B−chagasin	0.42	99.2	0.39	0.78
3K9M	cath_B−cyst_A	0.45	100.0	0.97	0.97
2NQD	cath_L−chagasin	0.38	99.6	0.97	0.99
3KSE	cath_L−cyst_A	0.34	99.4	0.63	0.63
3KFQ	cath_V−cyst_A	0.89	99.7	0.63	0.59
3E1Z	papain−chagasin	0.35	100.0	0.82	0.69
1STF	papain−cyst_B	1.20	96.5	0.97	0.82

^a^ Code identifier in the Protein Data Bank [37], SM_HM molecular models built by homology modelling using SWISS-MODEL [34]; RMSD, Root Mean Square Deviation computed from superpose evaluation of model SM_HM on PDB structure.

**Table 4:** Reference data of papain-like cysteine proteinases and their natural inhibitor in this study.

EC	Protease	Organism	ID	PDB ^a^	Inhibitor	Organism	ID
3.4.22.51	cruzipain	*T. cruzi*	P25779	1ME3 ^b^			
	congopain	*T. congolense*	Q26909				
3.4.22.B69	falcipain 2	*P. falciparum*	Q9N6S8	1YVB ^c^	Gg_cyst	*G. gallus*	P01038
	falcipain 2	*P. falciparum*	Q8I6U4	2OUL ^c^	chagasin	*T. cruzi*	Q966X9
	LMCP	*L. mexicana*	Q7JMY2				
3.4.22.B5	LMCP B	*L. mexicana*	P36400				
3.4.22.1	cath_B	*H. sapiens*	P07858	3CBK ^c^	chagasin	*T. cruzi*	Q966X9
3.4.22.1	cath_B	*H. sapiens*	P07858	3K9M ^c^	cyst_A	*H. sapiens*	P01040
3.4.22.16	cath_H	*H. sapiens*	P09668				
3.4.22.15	cath_L	*H. sapiens*	P07711	2NQD ^c^	chagasin	*T. cruzi*	Q966X9
3.4.22.15	cath_L	*H. sapiens*	P07711	3KSE ^c^	cyst_A	*H. sapiens*	P01040
3.4.22.43	cath_V	*H. sapiens*	O60911	3KFQ ^c^	cyst_A	*H. sapiens*	P01040
3.4.22.2	papain	*C. papaya*	P00784	3E1Z ^c^	chagasin	*T. cruzi*	Q966X9
3.4.22.2	papain	*C. papaya*	P00784	1STF ^c^	cyst_B	*H. sapiens*	P04080
3.4.22.6	chymo	*C. papaya*	P14080	1YAL ^b^			
3.4.22.30	caricain	*C. papaya*	P10056	1PPO ^b^			
					Cj_cyst	*C. japonica*	P81061
				3GAX ^b^	cyst_C	*H. sapiens*	P01034
				2C34 ^b^	LEIME	*L. mexicana*	Q868H1

EC, Enzyme Commission numbers; ^a^ structures obtained from RCSB PDB [37]; ^b^ crystal free protein structures; ^c^ crystal complex structures; ID, accession number on UniProt [42].

**Figure A1:**
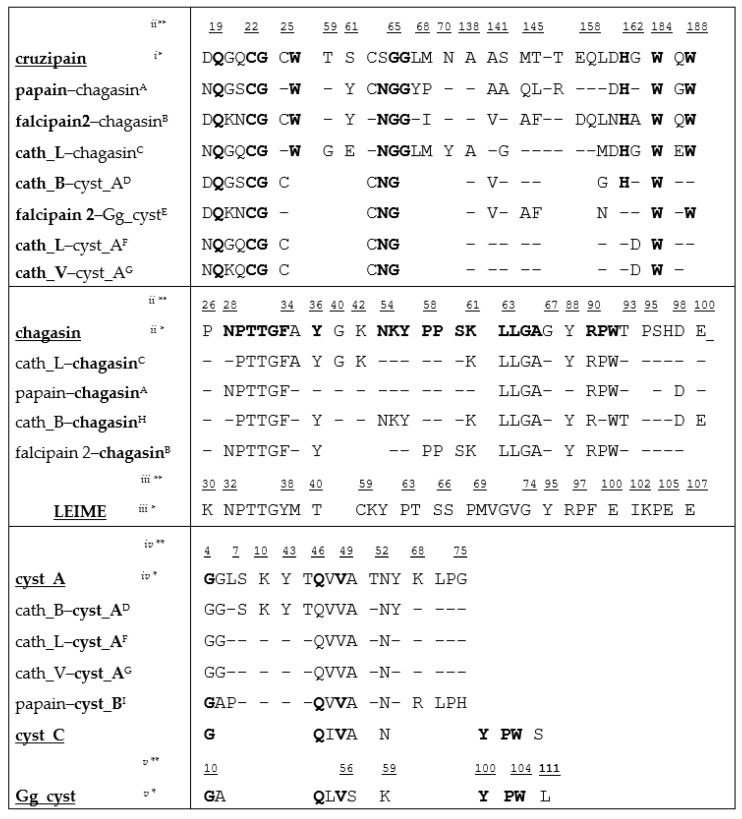
Identification of residues present in the CP to CPI interaction. Query: Cruzipain (PDB 1ME3), chagasin, LEIME (PDB 2C34) and cystatins. *, residues at binding site; **, numbering; *^i^*, cruzipain; *^ii^,* chagasin; *^iii^,* LEIME; *^iv^,* cyst_A; *^v^*, Gg_cyst. PDB: ^A^, 3E1Z; ^B^, 2OUL; ^C^ 2NQD; ^D^, 3K9M; ^E^, 1YVB; ^F^, 3KSE; ^G^, 3KFQ; ^H^, 3CBK; and ^I^, 1STF.

**Figure A2:**
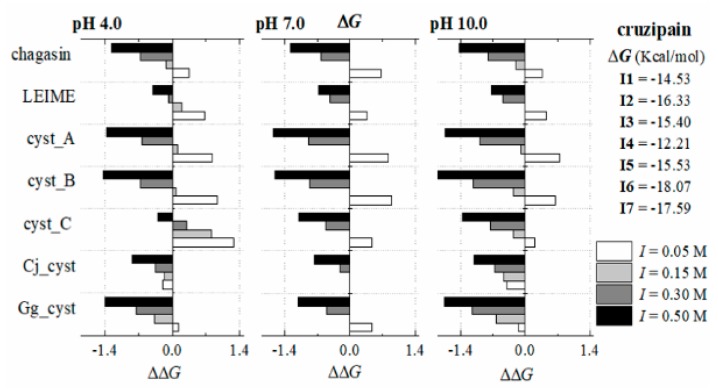
cruzipain−CPIs complex.

**Figure A3:**
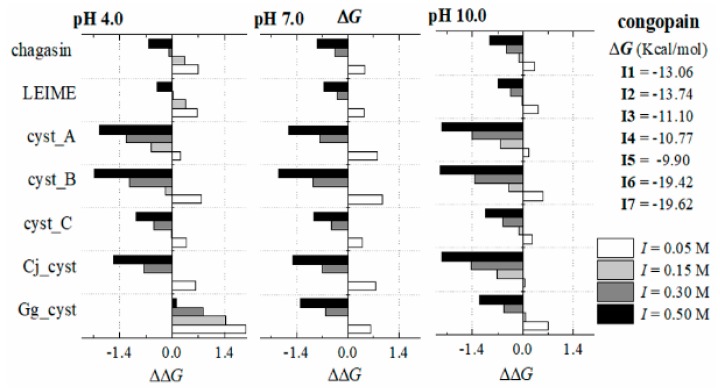
congopain−CPIs complex.

**Figure A4:**
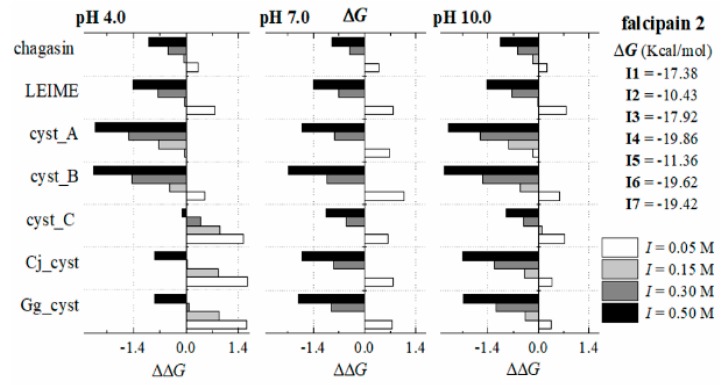
falcipain 2−CPIs complex.

**Figure A5:**
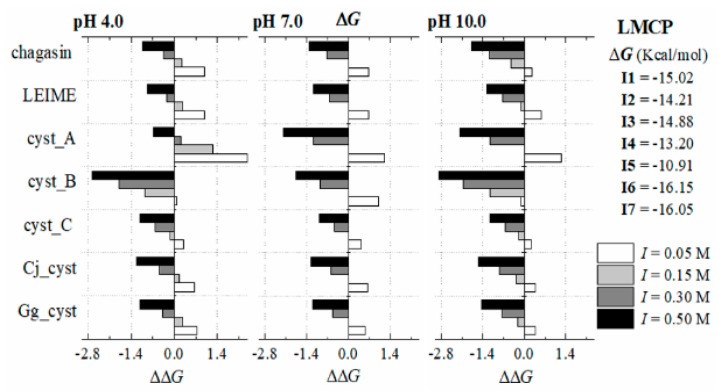
LMCP−CPIs complex.

**Figure A6:**
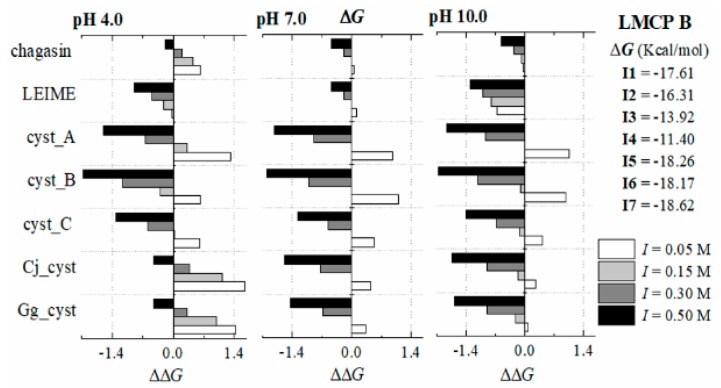
LMCP B−CPIs complex.

**Figure A7:**
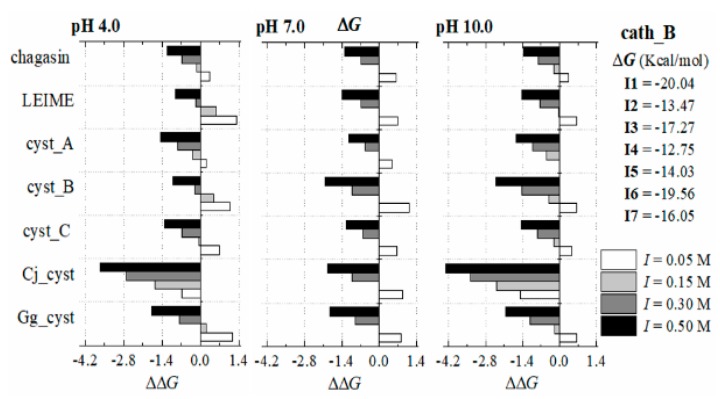
cath B−CPIs complex.

**Figure A8:**
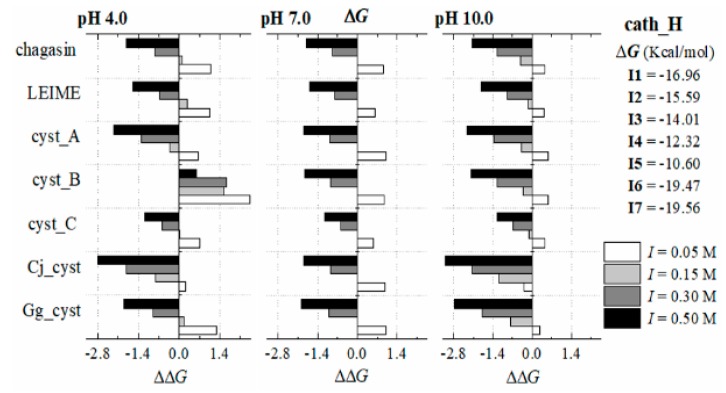
cath H−CPIs complex.

**Figure A9:**
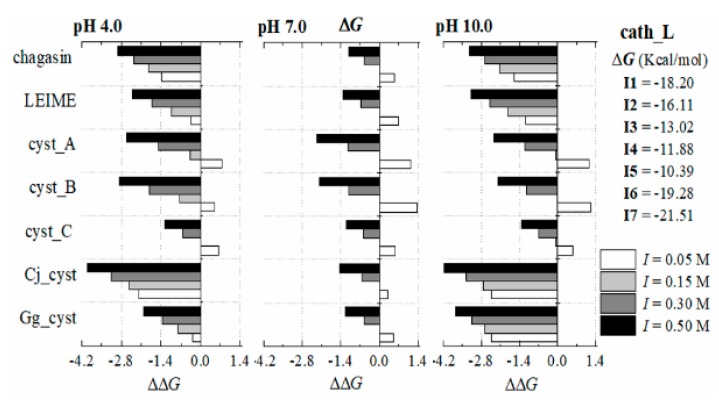
cath L−CPIs complex.

**Figure A10:**
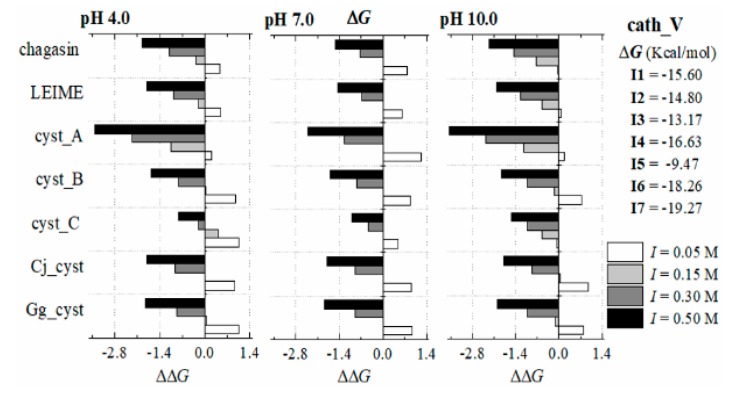
cath V−CPIs complex.

**Figure A11:**
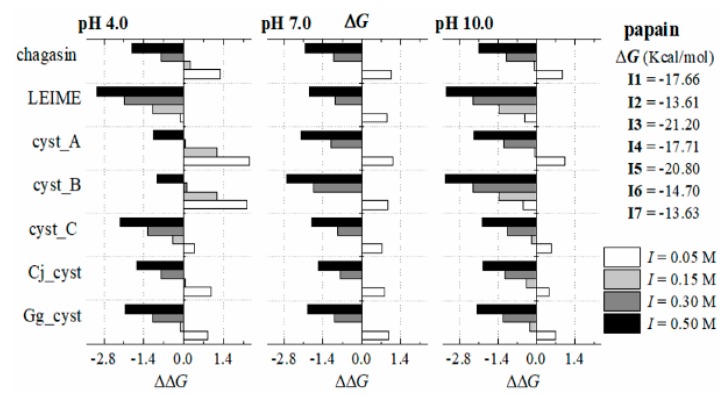
papain−CPIs complex.

## Abbreviations

Δ*G*_b_	binding energy
CP	cysteine proteinase
ICP	cysteine protease inhibitors
cath	cathepsin
QMQE	the global model quality estimation
cyst	ciystatin
*I*	ionic strength

## Appendix A

### Residues Present in the Interaction of CP to Natural ICP from Crystal Structures

The search for the interaction of the residues present in the interaction of CP to natural ICP from crystal structures (Figure A1) was made with the computational tool Find Interaction Partners and Binding Sites [46,47]. Protein structure in bold and underlined indicates the protein that was searched for in the database; those in bold represent the structures found in the templates registered in the database. The information contained in Table A1 allowed the identification of residues located in the protein−protein interaction site before constructing molecular models. Once the models were constructed, a 3D alignment of the homologous structures was carried out and the corresponding residues were located (e.g., Figure 2B,C).

## Appendix B

### Appendix B.1. Contribution of ΔH y ΔS to ΔG

Δ*G*_b_(T) were calculated respectively at 273, 313 and 323 K, *I* 0.15 M and pH 7.0 (see Materials and Methods). Then, Table A1 shows the result of the analysis carried out at 273 K, pH 7.0 and *I* 0.15 M.

**Table A1:** Profile of the contribution of enthalpy (Δ*H*) y entropy (−TΔ*S*) to ΔG (Kcal/mol) at 273 K, pH 7.0 and *I* 0.15 M.

ICP	chagasin		LEIME		cyst_A		cyst_B		cyst_C		Cj_cyst		Gg_cyst	
CP	Δ*H*	−TΔ*S*	Δ*H*	−TΔ*S*	Δ*H*	−TΔ*S*	Δ*H*	−TΔ*S*	Δ*H*	−TΔ*S*	Δ*H*	−TΔ*S*	Δ*H*	−TΔ*S*
E1	−20.3	5.8	−17.0	0.6	−19.0	3.5	−23.2	11.0	−18.5	2.9	−22.6	4.5	−22.8	5.1
E2	−16.6	3.6	−15.3	1.5	−18.4	7.3	−18.3	7.5	−12.5	2.6	−28.2	8.7	−26.8	7.2
E3	−22.1	4.8	−10.8	0.4	−20.2	2.3	−27.9	8.1	−13.3	2.0	−21.2	1.5	−20.9	1.5
E4	−21.7	6.7	−16.5	2.4	−24.1	9.2	−21.7	8.5	−15.7	4.8	−23.8	7.7	−23.5	7.5
E5	−18.1	0.5	−17.3	1.0	−21.6	7.7	−21.5	10.1	−23.7	5.5	−21.9	3.7	−22.0	3.4
E6	−21.6	1.5	−15.7	2.3	−22.0	4.8	−21.7	8.9	−16.4	2.4	−23.2	3.6	−23.0	7.0
E7	−22.9	5.9	−22.8	7.3	−22.5	8.4	−19.5	7.1	−14.0	3.4	−28.8	9.3	−23.9	4.3
E8	−19.8	1.6	−18.1	1.9	−24.5	11.4	−26.4	14.6	−14.6	4.3	−34.8	15.6	−31.8	10.2
E9	−19.8	4.2	−19.4	4.6	−26.3	13.1	−22.9	6.2	−13.1	3.6	−25.8	7.6	−27.7	8.4
E10	−22.8	5.1	−21.2	6.5	−30.0	8.7	−24.1	6.4	−26.5	5.7	−21.1	7.5	−22.6	9.0

CP, cysteine proteases: E1, cruzipain; E2, congopain; E3, falcipain 2; E4, LMCP; E5, LMCP B; E6, cath_B; E7, cath_H; E8, cath_L; E9, cath_V; E10, papain; ICP, cysteine protease inhibitors: chagasin, LEIME, cyst_A, cyst_B, cyst_C, Cj_cyst and Gg_cyst.

### Appendix B.2. Dependence of ΔGb (I, pH) at 298 K

Analysis of Δ*G*_b_ (*I*, pH) was performed on the variation of *I* (values of 0.05, 0.15, 3.0 and 5.0 M) and pH (values of 3.0, 7.0 and 10.0) at 298 K. In Figure A2, Figure A3, Figure A4, Figure A5, Figure A6, Figure A7, Figure A8, Figure A9, Figure A10 and Figure A11 we show the ΔΔ*G* calculated as ΔΔ*G* = (Δ*G*_b_ − Δ*G*), where Δ*G*_b_ (*I*, pH) at 273 K and Δ*G* reference were calculated respectively at 273 K, *I* 0.15 M and pH 7.0; data showed in box.

Figure A2, Figure A3, Figure A4, Figure A5, Figure A6, Figure A7, Figure A8, Figure A9, Figure A10 and Figure A11. Profile of Δ*G*_b_(I,pH). Δ*G*_b_, free energy; ΔΔ*G*, relative binding energy obtained from Δ*G*_b_ (binding energy) which was predicted at 278 K, pH 7.0 and *I* = 0.15 M. Binding inhibitor: I1, chagasin; I2, LEIME; I3, cyst_A; I4, cyst_B; I5, cyst_C; I6, Cj_cyst; I7, Gg_cyst.
